# NVP-CGM097, an HDM2 Inhibitor, Antagonizes ATP-Binding Cassette Subfamily B Member 1-Mediated Drug Resistance

**DOI:** 10.3389/fonc.2020.01219

**Published:** 2020-07-23

**Authors:** Meng Zhang, Xuan-Yu Chen, Xing-Duo Dong, Jing-Quan Wang, Weiguo Feng, Qiu-Xu Teng, Qingbin Cui, Jing Li, Xiang-Qi Li, Zhe-Sheng Chen

**Affiliations:** ^1^First Clinical College, Shandong University of Traditional Chinese Medicine, Jinan, China; ^2^Department of Pharmaceutical Sciences, College of Pharmacy and Health Sciences, St. John's University, Queens, NY, United States; ^3^College of Integrated Chinese and Western Medicine, Hebei Medical University, Shijiazhuang, China; ^4^College of Bioscience and Technology, Weifang Medical University, Weifang, China; ^5^Department of Breast Surgery, The Second Affiliated Hospital of Shandong First Medical University, Tai'an, China

**Keywords:** ABCB1, multidrug resistance, ABC transporter, NVP-CGM097, chemotherapy

## Abstract

Multidrug resistance (MDR) is a major challenge in the treatment of tumors. It refers to cancer cells become resistant to not only the therapeutic drug, but also cross-resistant to multiple drugs with distinct structures and mechanisms of action when they are exposed to a drug for a period of time. An essential mechanism of MDR is the aberrant expression and function of ATP-binding cassette (ABC) transporters. Therefore, blocking the function of ABC transporters has the therapeutic potential in reversing MDR. The hdm2 oncogene product, HDM2 (also known as MDM2), is an important negative regulator of the p53 tumor suppressor. NVP-CGM097 is an HDM2 inhibitor that can inhibit the proliferation of tumor cells and is currently under clinical trials. In this study, we evaluate whether NVP-CGM097 could reverse ABCB1-mediated MDR. The results of reversal experiment showed that NVP-CGM097 remarkably reversed ABCB1-mediated MDR but not ABCG2-mediated MDR. The results of Western blot and immunofluorescence suggested that the level of expression and subcellular localization of ABCB1 protein were not significantly altered by NVP-CGM097. Mechanism studies indicated that NVP-CGM097 could reverse ABCB1-mediated MDR by directly blocking the ABCB1-mediated drug efflux and raising the accumulation of chemotherapeutic drugs in cancer cells. ATPase analysis showed that low concentration NVP-CGM097 activates ABCB1 ATPase activity while high concentration NVP-CGM097 inhibited ABCB1-associated ATPase. Docking study indicated that NVP-CGM097 tended to bind to the inhibitory site, which led to slight but critical conformational changes in the transporter and reduced the ATPase activity. Overall, our study demonstrates that NVP-CGM097 can be used in conjunction with chemotherapeutic drugs to counteract MDR and improve the antitumor responses.

## Introduction

Malignant tumors pose a significant hazard to human life and each year the number of deaths caused by malignant tumors ranks first among all forms of diseases (1). Chemotherapy has been one of the important modalities for the treatment of malignant tumors. However, the phenomenon of multidrug resistance (MDR) of tumor cells is the main reason for the failure of chemotherapy (2, 3). MDR refers to the drug resistance of tumor cells to multiple antineoplastic drugs with different structures and mechanisms of actions (4). While the mechanism of MDR is complicated, the up-regulation and gain-of-function of ATP binding cassette (ABC) transporters is one of the important factors leading to MDR. ABC transporters constitute the largest superfamily of human cellular membrane transporters. It is divided into 7 subfamilies (ABCA~ABCG) based on gene homology and structural similarity (5). Among them, ABCB1 (P-gp/MDR1) and ABCG2 (BCRP/MXR/ABCP), as well as ABCCs (MRPs) have been extensively reported to be associated with MDR (6). Previous studies have reported that overexpression of ABCB1 is found in many tumors, such as gastric cancer (7), lung cancer (8), breast cancer (9), bladder cancer (10), prostate cancer (11), and so forth. It is also confirmed that the level of ABCB1 is strongly correlated with the chemosensitivity of tumors (12). Consequently, the overexpression of ABCB1 is an significant factor initiating the development of MDR and leading to the ineffectiveness of chemotherapy (13). Upon reaching the tumor cells, chemotherapeutic drugs first bind to ABCB1, then ATP hydrolysis generates energy for pumping the drugs out of the cells, decreasing the concentration of intracellular drugs, and therefore leading to drug resistance (14–16). Therefore, it is of great significance to develop a treatment option to reverse the MDR mediated by ABCB1.

HDM2 inhibitors are developed to inhibit HDM2-p53 interaction and thereby suppress p53 deterioration and reactivate the expression of wild-type p53. These inhibitors were under evaluation in clinical trials in adult patients with selected advanced solid tumors (17). Previous studies have shown that there is a synergistic effect between HDM2 inhibitors and ABCB1 substrates (18–20), but the mechanism related to the functional change of ABCB1 remains unknown. No direct interaction between HDM2 inhibitors and ABC transporters has been reported to date. In this study, we evaluated a novel HDM2-inhibitor NVP-CGM097 (17, 21, 22) against ABCB1-mediated MDR. Our results showed that NVP-CGM097 could reverse ABCB1-mediated MDR at non-cytotoxic concentration.

## Materials and Methods

### Chemicals

NVP-CGM097 was a gift from ChemieTek (Indianapolis, IN). DMEM, penicillin/streptomycin, and fetal bovine serum (FBS) were purchased from Corning Incorporated (Corning, NY, USA). Doxorubicin, paclitaxel, cisplatin, mitoxantrone, verapamil, 3-(4,5-dimethylthiazol-yl)-2,5-diphenyltetrazolium bromide (MTT), dimethylsulfoxide (DMSO), Triton X-100, the monoclonal mouse antibodies against ABCB1 (clone F4, Cat # SAB4200775), were products from Sigma-Aldrich (St. Louis, MO, USA). The Horseradish peroxidase (HRP)-conjugated rabbit anti-mouse IgG secondary antibody (Cat # 7076S, lot #: 32) was obtained from Cell Signaling Technology Inc. (Danvers, MA, USA). The Alexa Fluor 488 conjugated goat anti-mouse IgG cross-adsorbed secondary antibody (2 mg/mL, Cat # A32723) and the GAPDH loading control monoclonal mouse antibody (GA1R) (1 mg/mL, Cat # MA5-15738, lot #: SA247966) were purchased from Thermo Fisher Scientific Inc. (Rockford, IL, USA).

### Cell Lines and Cell Culture

The human epidermoid carcinoma KB-3-1 cell line and its colchicine-selected ABCB1-overexpressing KB-C2 cell line (23, 24), the human colon cancer SW620 cell line and its doxorubicin-selected ABCB1-overexpressing SW620/Ad300 cell line were used for ABCB1 reversal study (25). The non-small cell lung cancer (NSCLC) cell line NCI-H460 and its mitoxantrone-selected ABCG2-overexpressing NCI-H460/MX20 cell line were used for ABCG2 reversal study (26). HEK293/pcDNA3.1 and the ABCB1-transfected HEK293 cells (HEK293/ABCB1) were human embryonic kidney HEK293 cells transfected with empty vector pcDNA3.1 and full length ABCB1, respectively, (27). All of the drug-resistant samples were cultivated for at least 2 weeks before use in a medium without drugs.

### Cytotoxicity Assay

The cells in logarithmic growth phase were collected and seeded in 96-well plates with a concentration of 5,000 to 6,000 cells per well. After the cells were incubated overnight, NVP-CGM097 and a parallel control inhibitor of transporter were added 2 h prior. A chemotherapeutic drug was then added into the designated wells by in a concentration range. After 72 h of culture, 20 μl of MTT reagent (0.4 mg/ml) was added to each well and incubated in the incubator for 4 h, then the drug solution was discarded and DMSO solution was added. After gently oscillating 10 min on the microplate oscillator, the absorbance was measure at 570 nm in a Spectrophotometer (Fisher Sci., Fair Lawn, NJ, USA) with accuSkan™ GO UV/Vis Microplate (Fisher Sci., Fair Lawn, NJ, USA).

### Western Blotting Analysis

The Western blotting protocol has been stated in previous study (28). Cells were treated with or without NVP-CGM097 (3 μM) for different time periods (0, 24, 48, 72 h), and cells were lysed after being washed twice with ice-cold PBS. Concentrations of proteins were calculated using Pierce™ BCA Protein Assay Kit (Thermo Scientific, Rockford, IL, USA). The total protein was then resolved by gel electrophoresis (25 μg/lane). The protein was transferred to the activated PVDF membrane and blocked by 5% non-fat milk. The membrane was incubated in monoclonal primary mouse antibodies against GAPDH (1:1000) or ABCB1 (1:1000) overnight, and then followed by incubation with HRP-conjugated rabbit anti-mouse IgG secondary antibody (1:1000) for 1 h. ECL chemiluminescence kit was used and the signal was captured by X-ray film.

### Immunocytochemistry

Immunocytochemistry was carried out as documented in the previous researches (29). KB-3-1 and KB-C2 cells (1 × 10^4^/well) were seeded into 24-well plates and incubated for 24 h, followed by incubation with 0.3 μM NVP-CGM097 for 0, 24, 48, and 72 h. The fixation was performed for 15 min with 4% formaldehyde, followed by permeabilization for 15 min with 0.25% Triton X-100, then blocked for 1 h with 6% BSA. The cells were then treated with monoclonal primary mouse antibody against ABCB1 (1:1000) overnight at 4°C, then with secondary goat anti-mouse IgG antibody combined with Alexa Fluor 488. DAPI was used to counterstain the nuclei. A Nikon TE-2000S microscope (Nikon Instruments Inc., Melville, NY, USA) was used to conduct microscopy.

### ATPase Assay

The vanadate-sensitive ABCB1 ATPase activity is examined using the Pgp-Glo™ assay system (Promega, Madison, WI, USA) following the manufacturer's protocol. In brief, an equal amount of membrane vesicles was incubated in the ATPase assay buffer with or without sodium orthovanadate (Na_3_VO_4_) and Mg-ATP at 37°C. The amount of inorganic phosphates was determined by a colorimetric method as previously described (30).

### [^3^H]-Paclitaxel Accumulation and Efflux Assay

The substrate [^3^H]-paclitaxel was used to perform the drug accumulation and efflux assay to investigate the reversal mechanism of NVP-CGM097 as previously reported (31, 32). For accumulation assay, KB-3-1 and KB-C2 cells (1 × 10^5^) were seeded in 24-well plates with overnight incubation. Different concentrations of NVP-CGM097 (0, 1, 3 μM) and verapamil (3 μM) were added in designated wells for 2 h following incubation with [^3^H]-paclitaxel for another 2 h at 37°C. After washing with ice-cold PBS, cells were lysed and collected in 5 ml scintillation fluid. For efflux assay, NVP-CGM097 (0, 1, 3 μM) and verapamil (3 μM) were added in designated wells for 2 h following incubation with [^3^H]-paclitaxel for another 2 h at 37°C. Subsequently, supernatant was removed and fresh drug-free medium with or without an inhibitor was added. The lysed cells were collected at various time points (0, 30, 60, and 120 min) in 5 ml scintillation fluid. Radioactivity of cells was determined by the Packard TRI-CARB 1900CA liquid scintillation analyzer (Packard Instrument, Downers Grove, IL, USA) (33).

### Molecular Docking of NVP-CGM097 With Human ABCB1 Models

As previously mentioned, the NVP-CGM097 3-D structure was built to dock simulation with a human ABCB1 model (34). Human ABCB1 protein model 6QEX (paclitaxel bound) and 6QEE (zosuquidar bound) were obtained from RCSB Protein Data Bank. Both models are inward-facing human ABCB1 with a resolution of 3.6 Å (6QEX) or 3.9 Å (6QEE) (35). Calculations of the docking were achieved in AutoDock Vina (version 1.1.2) (36). AutoDockTools (ADT, version 1.5.4) was used to add hydrogen atoms and partial charges. Docking grid center coordinates were determined from the bound ligands provided in PDB files. Docking simulation and the receptor/ligand configuration was conducted using default settings. For further study and visualization, the top-scoring pose (sort by affinity score: kcal/mol) was identified.

### Statistical Analysis

The results in this study are presented as mean ± SD and analyzed using one-way ANOVA followed by the Dunnett's test. All data was generated from at least three independent experiments with triplicates or duplicates.

## Results

### NVP-CGM097 Sensitized Chemotherapeutic Drugs in ABCB1-Overexpressing Cells

**Figure 1:**
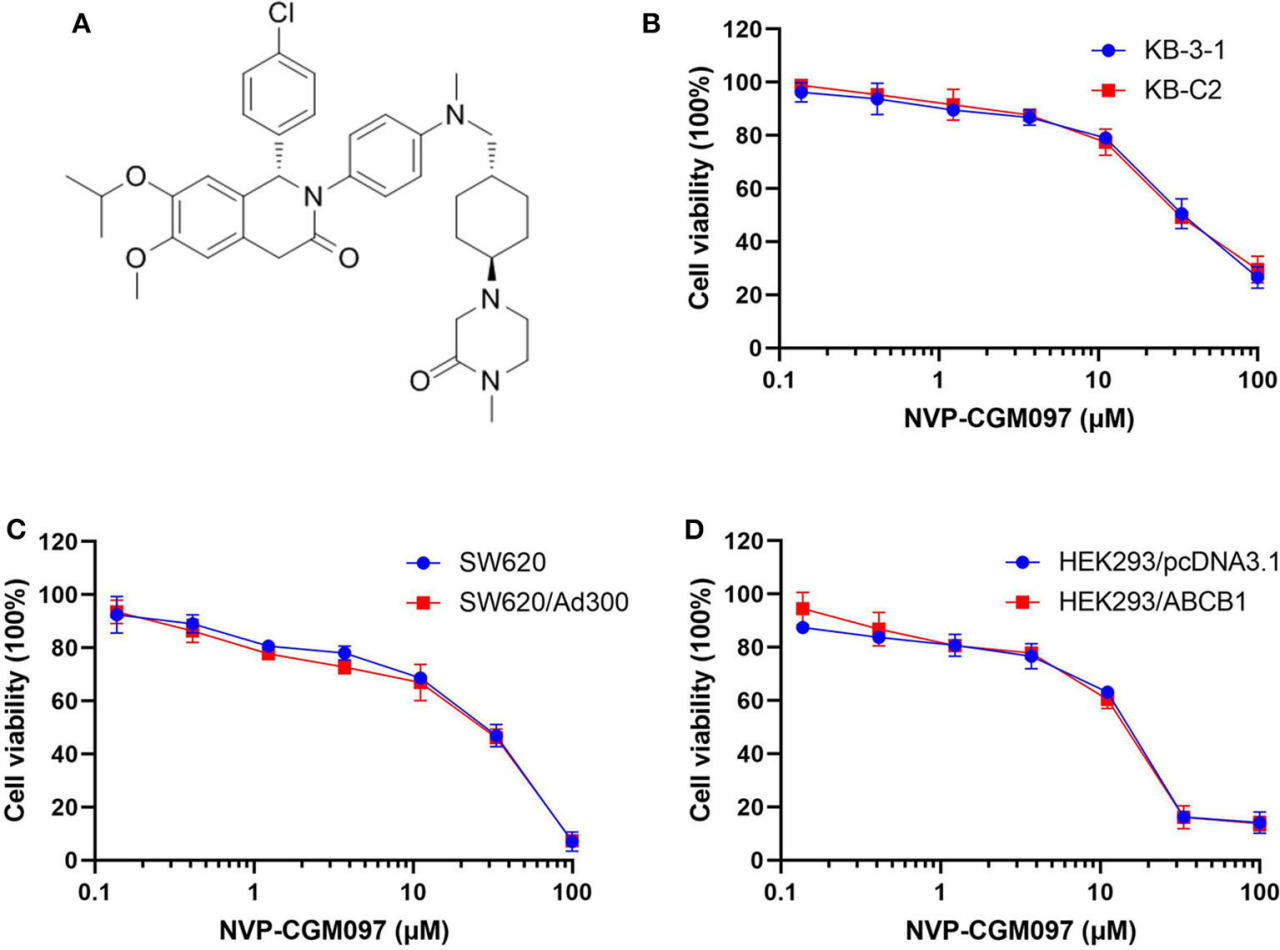
Chemical structure of NVP-CGM097 and concentration-dependent viability curves for parental and ABCB1-overexpressing cells incubated with NVP-CGM097. **(A)** Chemical structure of NVP-CGM097. **(B)** Cytotoxicity curves for KB-3-1 and KB-C2 cells incubated with NVP-CGM097 for 72 h. **(C)** Cytotoxicity curves for SW620 and SW620/Ad300 cells incubated with NVP-CGM097 for 72 h. **(D)** Cytotoxicity curves for HEK293/pcDNA3.1 and HEK293/ABCB1 cells incubated with NVP-CGM097 for 72 h. The cell viability was determined by MTT assay. Data are expressed as mean ± SD, and representative of three independent experiments in triplicate are shown.

In this study, we used NCI-H460 and its drug-selected ABCG2-overexpressing cell line NCI-H460/MX20, KB-3-1 and its drug-selected ABCB1-overexpressing cell line KB-C2, SW620 cells and its drug-selected ABCB1-overexpressing cell line SW620/Ad300. Considering that drug selected cell lines have multiple factors affected their sensitivity to drugs, ABCG1 gene-transfected HEK293 cells were used to further validate the results. The cytotoxicity of NVP-CGM097 in these cell lines was first evaluated. The IC_50_ and IC_20_ of NVP-CGM097 for KB-3-1 were 44.03 and 13.53 μM, respectively, for KB-C2 were 45.54 and 11.24 μM, respectively, for SW620 were 25.20 and 4.23 μM, respectively, for SW620/Ad300 were 17.05 and 3.82 μM, respectively, for HEK293/pcDNA3.1 were 14.36 and 3.99 μM, respectively, for HEK293/ABCB1 were 14.57 and 4.02 μM, respectively. We selected the concentrations in which no more than 20% cell proliferation inhibition was observed as the concentrations used in the drug combination assays. As shown in Figure 1, we chose 1 and 3 μM as non-toxic concentrations in the following experiments. As shown in Table 1, co-treatments of NVP-CGM097 significantly decreased the IC_50_ values of doxorubicin and paclitaxel in KB-C2 and SW620/Ad300 cells as compared to the control group. In addition, the potencies of doxorubicin and paclitaxel in HEK293/ABCB1 cells were substantially increased by NVP-CGM097, compared with that in the empty vector transfected HEK293/pcDNA3.1 cells (Table 2). These results indicate that NVP-CGM097 sensitized chemotherapeutic drugs in ABCB1-overexpressing cells. In addition, in ABCG2 overexpressed NCI-H460/MX20 cells, NVP-CGM097 did not alter the sensitivity against mitoxantrone, an ABCG2 substrate (Table 3). These results suggest that NVP-CGM097 can restore the chemo-sensitivity in ABCB1 overexpressing cells. Verapamil was used as a positive control in this study as a known ABCB1 inhibitor, Ko143 was used as a positive control inhibitor of ABCG2. In addition, cisplatin is used as a negative control anticancer drug as it is a non-substrate of both ABCB1 and ABCG2.

**Table 1:** NVP-CGM097 sensitized ABCB1-substrate-selected resistant cells to ABCB1 substrates.

**Treatment**	**IC_**50**_ ± SD^a^ (RF^b^) (μM)**			
	**SW620**	**SW620/Ad300**	**KB-3-1**	**KB-C2**
Doxorubicin	0.232 ± 0.034 (1.00)	19.687 ± 0.711 (84.86)	0.011 ± 0.001 (1.00)	1.575 ± 0.477 (143.18)
+NVP CGM097 (1 μM)	0.307 ± 0.033 (1.32)	6.831 ± 1.030 (29.44)^*^	0.011 ± 0.002 (1.00)	0.344 ± 0.082 (31.27)^*^
+NVP CGM097 (3 μM)	0.215 ± 0.018 (0.92)	1.462 ± 0.212 (6.30)^*^	0.012 ± 0.001 (1.09)	0.047 ± 0.007 (4.27)^*^
+Verapamil (3 μM)	0.222 ± 0.022 (0.95)	1.511 ± 0.087 (6.51)^*^	0.013 ± 0.001 (1.18)	0.065 ± 0.027 (5.91)^*^
Paclitaxel	0.220 ± 0.01 (1.00)	20.95 ± 1.47 (95.22)	0.016 ± 0.002 (1.00)	1.897 ± 0.133 (118.56)
+NVP CGM097 (1 μM)	0.196 ± 0.02 (0.89)	3.453 ± 0.59 (15.70)^*^	0.015 ± 0.002 (0.94)	0.378 ± 0.129 (23.63)^*^
+NVP CGM097 (3 μM)	0.237 ± 0.02 (1.08)	0.843 ± 0.32 (3.83)^*^	0.014 ± 0.001 (0.88)	0.021 ± 0.018 (1.31)^*^
+Verapamil (3 μM)	0.206 ± 0.07 (0.94)	0.957 ± 0.12 (4.35)^*^	0.015 ± 0.001 (0.94)	0.032 ± 0.002 (2.00)^*^
Cisplatin	1.021 ± 0.112 (1.00)	1.109 ± 0.113 (1.09)	1.259 ± 0.104 (1.00)	1.091 ± 0.071 (0.87)
+NVP CGM097 (1 μM)	1.093 ± 0.093 (1.07)	1.237 ± 0.101 (1.21)	1.206 ± 0.196 (0.96)	1.173 ± 0.091 (0.93)
+NVP CGM097 (3 μM)	1.236 ± 0.094 (1.21)	1.117 ± 0.122 (1.09)	1.096 ± 0.016 (0.87)	1.242 ± 0.109 (0.99)
+Verapamil (3 μM)	0.918 ± 0.098 (0.90)	1.102 ± 0.093 (1.07)	1.689 ± 0.078 (1.34)	1.342 ± 0.137 (1.07)

a *IC50 values were determined by MTT assay as described in “Materials and Methods,” and were obtained from three independent experiments in triplicate*.

b *Resistance fold (RF) was calculated from dividing the IC50 values of resistant cells (SW620/Ad300 and KB-C2) by the IC50 of parental cells (SW620 and KB-3-1) in the presence or absence of NVP-CGM097 or positive control inhibitor*.

* *p < 0.05 vs. group treated with antineoplastic drug only*.

**Table 2:** NVP-CGM097 sensitized ABCB1-gene-transfected cells to ABCB1 substrates.

**Treatment**	**IC_**50**_ ± SD^a^ (RF^b^)(μM)**	
	**HEK293/pcDNA3.1**	**HEK293/ABCB1**
Doxorubicin	0.012 ± 0.004 (1.00)	0.289 ± 0.081 (24.08)
+NVP CGM097 (1 μM)	0.014 ± 0.004 (1.17)	0.123 ± 0.017 (10.25)^*^
+NVP CGM097 (3 μM)	0.017 ± 0.014 (1.42)	0.043 ± 0.004 (3.58)^*^
+Verapamil (3 μM)	0.015 ± 0.003 (1.25)	0.066 ± 0.002 (5.50)^*^
Paclitaxel	0.016 ± 0.004 (1.00)	0.865 ± 0.260 (54.06)
+NVP CGM097 (1 μM)	0.020 ± 0.009 (1.25)	0.211 ± 0.028 (13.19)^*^
+NVP CGM097 (3 μM)	0.014 ± 0.004 (0.88)	0.025 ± 0.002 (1.56)^*^
+Verapamil (3 μM)	0.019 ± 0.001 (1.19)	0.030 ± 0.002 (1.88)^*^
Cisplatin	1.228 ± 0.093 (1.00)	1.184 ± 0.133 (0.96)
+NVP CGM097 (1 μM)	1.364 ± 0.098 (1.11)	1.213 ± 0.067 (0.99)
+NVP CGM097 (3 μM)	1.104 ± 0.164 (0.90)	1.025 ± 0.039 (0.83)
+Verapamil (3 μM)	1.705 ± 0.187 (1.39)	0.972 ± 0.131 (0.80)

a *IC50 values were determined by MTT assay as described in “Materials and Methods,” and were obtained from three independent experiments in triplicate*.

b *Resistance fold (RF) was calculated from dividing the IC50 values of resistant cells (HEK293/ABCB1) by the IC50 of parental cells (HEK293/pcDNA3.1) in the presence or absence of NVP-CGM097 or positive control inhibitor*.

* *p < 0.05 vs. group treated with antineoplastic drug only*.

**Table 3:** NVP-CGM097 did not affect ABCG2-mediated MDR.

**Treatment**	**IC_**50**_ ± SD^a^ (RF^b^)(μM)**	
	**NCI-H460**	**NCI-H460/MX20**
Mitoxantrone	0.035 ± 0.002 (1.00)	5.567 ± 0.011 (159.05)
+NVP CGM097 (1 μM)	0.029 ± 0.001 (0.82)	5.762 ± 0.177 (164.63)
+NVP CGM097 (3 μM)	0.026 ± 0.023 (0.74)	5.538 ± 0.039 (158.23)
+Ko143(3 μM)	0.025 ± 0.002 (0.71)	0.404 ± 0.005 (11.54)^*^
Cisplatin	2.871 ± 0.012 (1.00)	2.698 ± 0.333 (0.94)
+NVP CGM097 (1 μM)	2.602 ± 0.074 (0.91)	2.346 ± 0.174 (0.82)
+NVP CGM097 (3 μM)	2.611 ± 0.05 (0.91)	3.101 ± 0.039 (1.08)
+KO143 (3 μM)	2.903 ± 0.063 (1.01)	2.212 ± 0.313 (0.77)

a *IC50 values were determined by MTT assay as described in “Materials and Methods,” and were obtained from three independent experiments in triplicate*.

b *Resistance fold (RF) was calculated from dividing the IC50 values of resistant cells (NCI-H460/MX20) by the IC50 of parental cells (NCI-H460) in the presence or absence of NVP-CGM097 or positive control inhibitor*.

* *p < 0.05 vs. group treated with antineoplastic drug only*.

### NVP-CGM097 Did Not Affect the Level of Expression and Subcellular Localization of ABCB1 Protein

**Figure 2:**
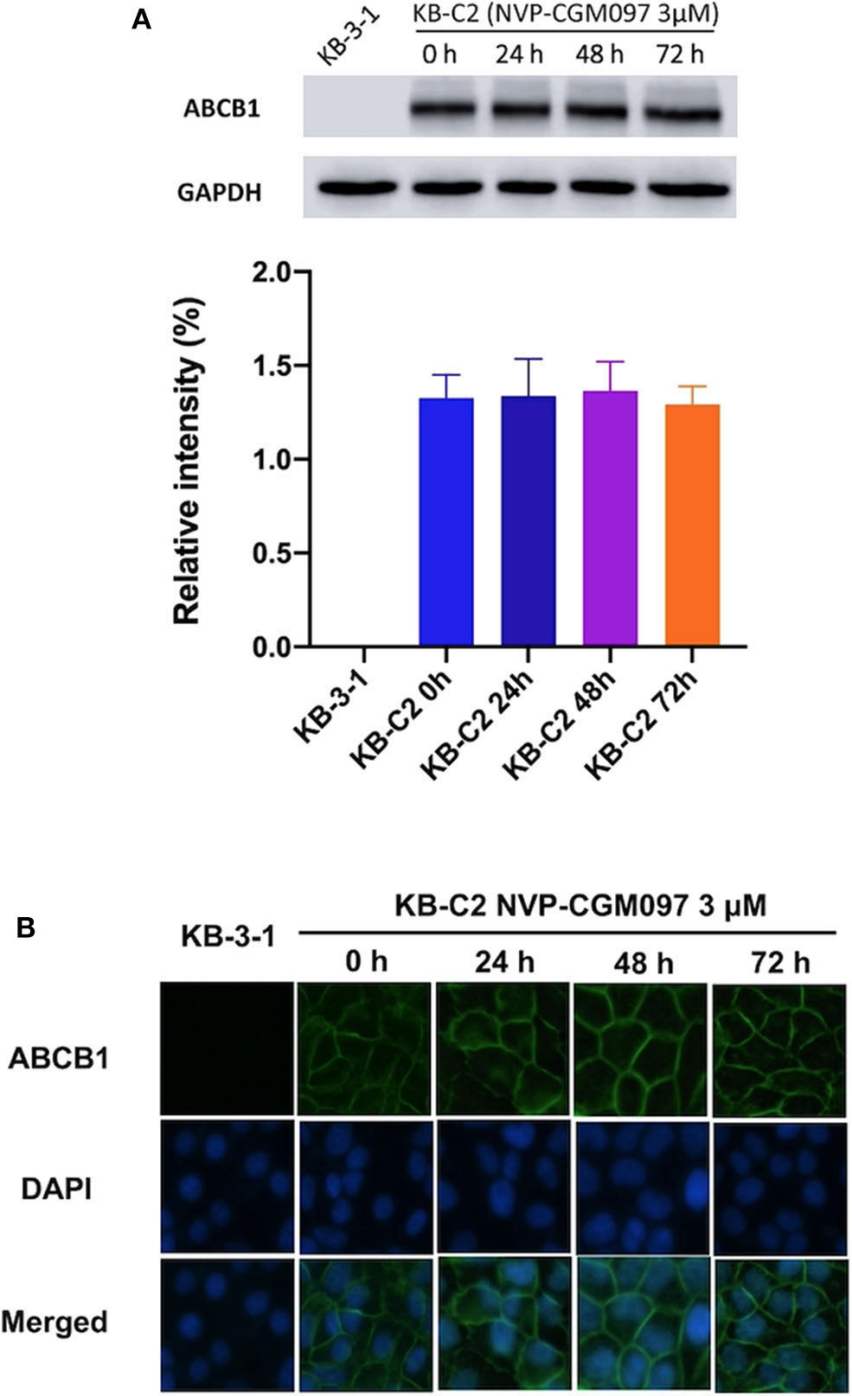
The effect of NVP-CGM097 on the protein expression and subcellular localization of ABCB1 transporters. **(A)** Detection of ABCB1 expression in KB-C2 cells incubated with 3 μM of NVP-CGM097 for 0, 24, 48, and 72 h. **(B)** Sub-cellular localization of ABCB1 expression in KB-C2 cells incubated with 3 μM of NVP-CGM097 for 0, 24, 48, and 72 h. Data are mean ± SD, representative of three independent experiments. Green: ABCB1. Blue: DAPI counterstains the nuclei. KB-3-1 represented the control group.

Down-regulation of ABC transporters or relocation of them from the cell membranes to the cytoplasm has been reported to minimize MDR in cancer cells (33). To evaluate those possible mechanisms, we performed Western blotting and immunofluorescence assays on NVP-CGM097-treated ABCB1-overexpresing cells. After incubation with 3 μM NVP-CGM097 for 0, 24, 48, and 72 h, the expression of ABCB1 (170 kDa) in KB-C2 cells did not significantly change (Figure 2A). Besides, the ABCB1 transporter was retained on the cell membranes after the treatment with NVP-CGM097 for 0, 24, 48, and 72 h (Figure 2B). These results suggest that NVP-CGM097 treatment up to 72 h could not alter the expression and localization of ABCB1 protein. Whether NVP-CGM097 can affect ABCB1 expression level or localization after longer time treatment warrants further investigation.

### NVP-CGM097 Increased the Intracellular Accumulation of [^3^H]-Paclitaxel in ABCB1-Overexpressing Cells

**Figure 3:**
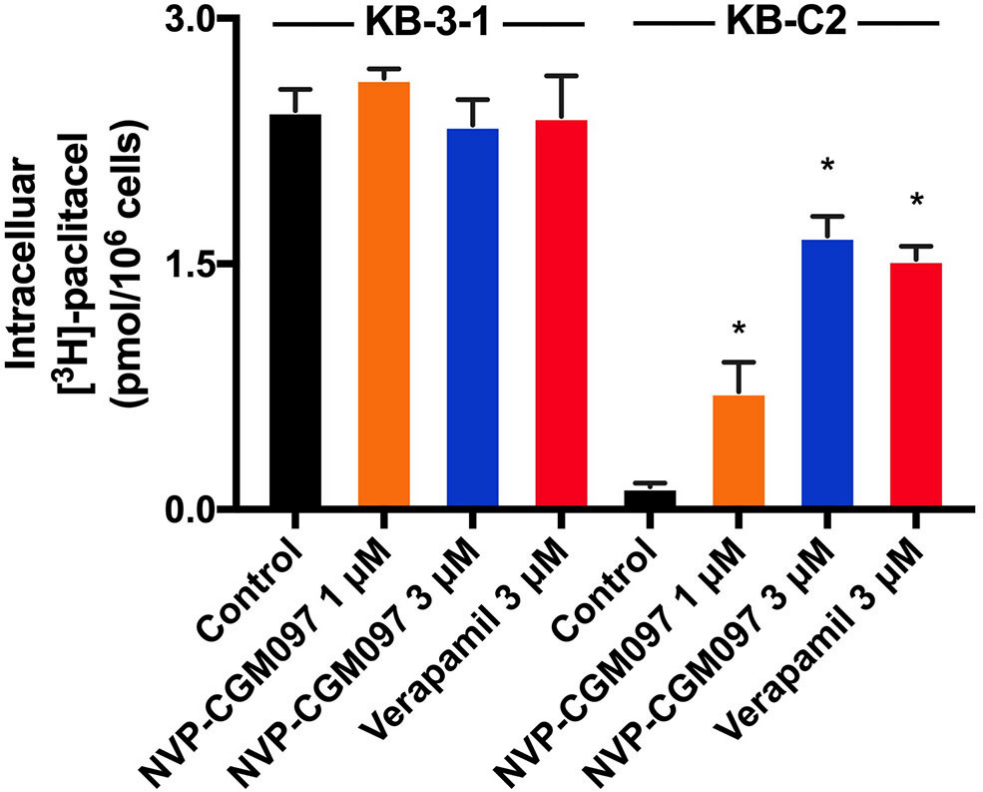
NVP-CGM097 increased the intracellular [^3^H]-drug accumulation in cancer cells ABCB1-overexpressing. The effect of NVP-CGM097 on the accumulation of [^3^H]-paclitaxel in KB-3-1 and KB-C2 cells. Verapamil (3 μM) were used as positive controls for ABCB1-overexpressing cells. Data are mean ± SD, representative of three independent experiments. ^*^p < 0.05, compared with control group.

To better understand how NVP-CGM097 antagonizes ABCB1-mediated MDR, the [^3^H]-paclitaxel accumulation assay was conducted. Our findings show that 3 μM of NVP-CGM097 significantly increases [^3^H]-paclitaxel intracellular accumulation in KB-C2 cells. The increasing effect of 3 μM of NVP-CGM097 is similar to the effect of 3 μM of verapamil in ABCB1-overexpressing KB-C2 cells (Figure 3). Besides, NVP-CGM097 did not affect the accumulation of parental KB-3-1 cells. These results indicate that NVP-CGM097 increases the intracellular accumulation of [^3^H]-paclitaxel in ABCB1-mediated MDR cells.

### NVP-CGM097 Inhibited the Efflux of [^3^H]-Paclitaxel in ABCB1-Overexpressing Cells

**Figure 4:**
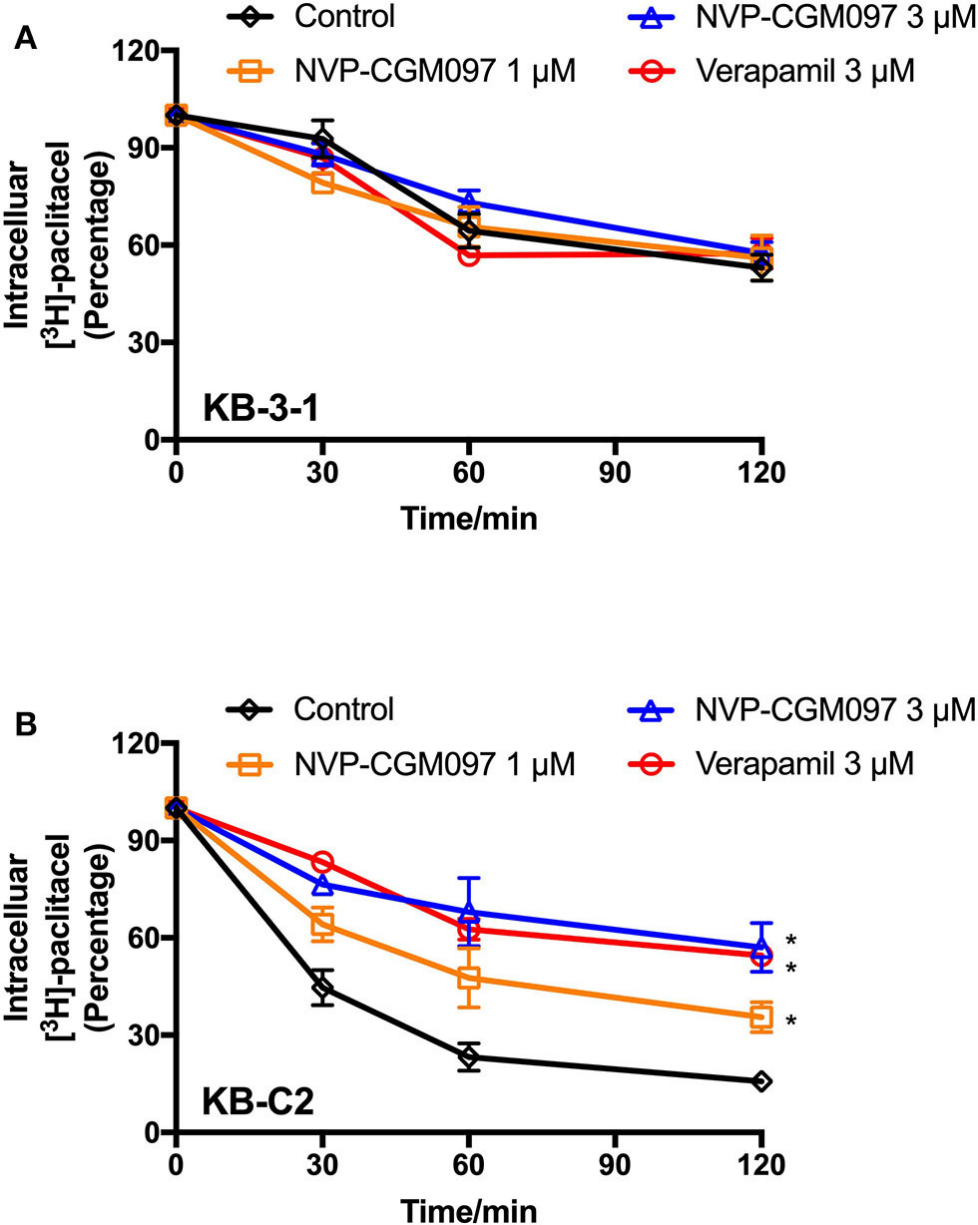
NVP-CGM097 inhibited the efflux function of ABCB1 transporters. **(A,B)** The effects of NVP-CGM097 on efflux of [^3^H]-paclitaxel in KB-3-1 and KB-C2 cells. Data are mean ± SD, representative of three independent experiments. ^*^p < 0.05, compared with control group.

Since there are multiple factors (either increase drug uptake or decrease drug efflux) that can result in increased paclitaxel accumulation, we explored whether NVP-CGM097 can inhibit the efflux function of ABCB1. The efflux assay was performed to further examine the dynamic process of resistant cancer cells re-sensitization by treatment of NVP-CGM097. As shown in Figure 4, NVP-CGM097 did not alter the [^3^H]-paclitaxel efflux in parental KB-3-1 cells. However, the [^3^H]-paclitaxel efflux activity was significantly decreased by treatment of NVP-CGM097. The obtained results showed that NVP-CGM097 can block the efflux activity of ABCB1-overexpressing cells, therefore, increasing intracellular paclitaxel accumulation.

### The Effect of NVP-CGM097 on ABCB1 ATPase Activities

**Figure 5:**
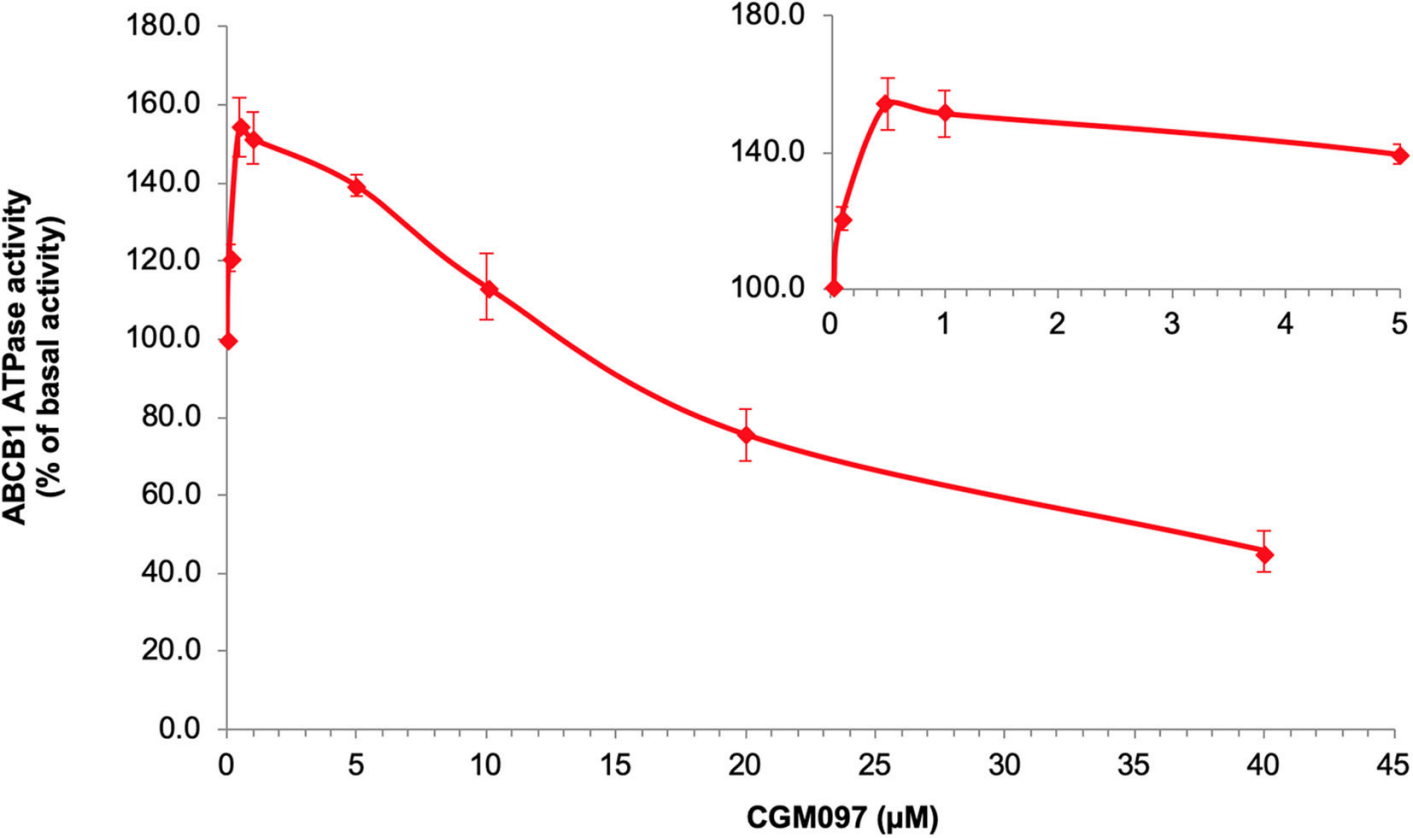
NVP-CGM097 stimulate first and then inhibit the ATPase activity of ABCB1. Effect of various concentrations of NVP-CGM097 on the ATPase activity of ABCB1. The inset graphs illustrate the effect of 0–4 μM NVP-CGM097 on the ATPase activity of ABCB1. Data are mean ± SD, representative of three independent experiments.

We tested ABCB1-mediated ATP hydrolysis in membrane vesicles after incubation at various NVP-CGM097 concentrations (0–40 μM), to further assess the influence of NVP-CGM097 on ABCB1 ATPase operation. According to the result (Figure 5), NVP-CGM097 stimulated the ABCB1-associated ATPase to a maximum of 154.3% of the basal activity at concentration range of 0–1 μM and NVP-CGM097's stimulatory impact reached a limit of 50 per cent (EC50) at 0.45 μM. In addition, at higher concentration, NVP-CGM097 showed inhibitory effect to the ATPase of ABCB1.

### Docking Simulation of NVP-CGM097 in the Drug-Binding Pocket of Human ABCB1

**Figure 6:**
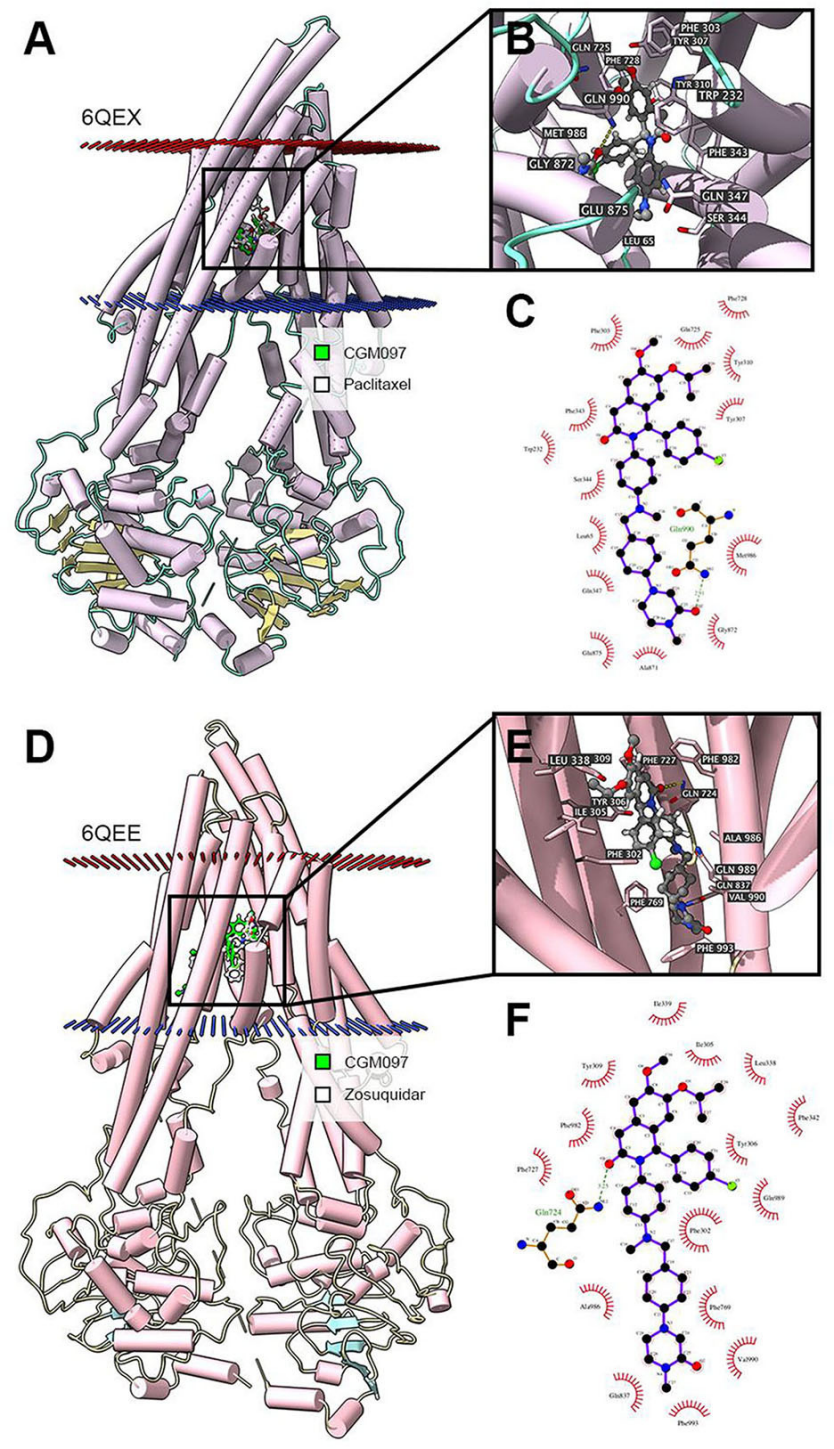
Interaction between NVP-CGM097 and human ABCB1 protein. **(A)** Overview of paclitaxel and the best-scoring pose of NVP-CGM097 in the drug binding pocket of ABCB1 protein (6QEX). Cytoplasm membrane was depicted as dotted planes where red or blue plane indicate extracellular or intracellular side, respectively. ABCB1 was displayed as colored tubes and ribbons. NVP-CGM097 and paclitaxel were displayed as colored sticks. Carbon: lime green (NVP-CGM097) or white (paclitaxel); oxygen: red; nitrogen: blue; chloride: green. **(B)** Details of interactions between NVP-CGM097 and ABCB1 (6QEX) binding pocket. ABCB1 was displayed as purple tubes. Important residues were displayed as colored sticks (carbon: purple; oxygen: red; nitrogen: blue). NVP-CGM097 was displayed as colored sticks (carbon: gray; oxygen: red; nitrogen: blue; chloride: green). Hydrogen bonds were displayed as yellow dash lines. **(C)** 2D NVP-CGM097-P-gp (6QEX) interaction. Important amino acids were displayed as red arcs, and green dash line with number indicates hydrogen bond with bond length. Carbon: black; oxygen: red; nitrogen: blue; chloride: green. **(D)** Overview of zosuquidar and the best-scoring pose of NVP-CGM097 in the drug binding pocket of ABCB1 protein (6QEE). **(E)** Details of interactions between NVP-CGM097 and ABCB1 (6QEE) binding pocket. Hydrogen bonds were displayed as yellow dash lines. **(F)** 2D NVP-CGM097-P-gp (6QEE) interaction. Color codes are same as **(C)**.

In the above result of ATPase assay, NVP-CGM097 displayed stimulating effect at lower concentration while inhibitory effect at higher concentration on ATPase. We applied docking simulation in both the ATPase-stimulator (substrate) binding site (6QEX) and the ATPase-inhibitor binding site (6QEE) of ABCB1 protein. The results showed that NVP-CGM097 docked into the substrate and inhibitory binding site with an affinity score of −8.5 kcal/mol and −10.2 kcal/mol, respectively. Details of ligand-receptor interaction was displayed in Figure 6. The primary factor leading to the binding of NVP-CGM097 to the ABCB1 protein for substratum binding sites is through hydrophobic interactions. NVP-CGM097 is positioned and stabilized in the hydrophobic cavity formed by Tyr310, Tyr307, Ile306, Phe303, Ile340, Phe343, and Ala871. Additionally, the oxopiperazin group of NVP-CGM097 was stabilized by a hydrogen bond formed with Gln990. For inhibitor binding site, the oxodihydroisoquinoline group of NVP-CGM097 was stabilized via hydrogen bond with Gln724. Besides, NVP-CGM097 was also stabilized by hydrophobic interaction in the cavity formed by Phe302, Ile305, Tyr309, Tyr306, Ile339, Phe342, Phe769, Phe993, and Ala986.

## Discussion

MDR is the main reason for the failure of drug treatment in cancer therapy. One of the most fundamental reasons of MDR is the overexpression of ABCB1 (13). Screening ABCB1 inhibitors is one of the strategies to reverse ABCB1-mediated MDR. ABCB1 inhibitors reverse MDR by inhibiting ABCB1 transporter efflux activity or by down-regulating ABCB1 protein expression, reducing the efflux of chemotherapeutic drugs and therefore increasing drug accumulation in tumor cells (37, 38). The common reasons for lack of ABCB1-reversal agents involve low level drug selectivity, weak targeting, excessive initial dose, large adverse reactions, and unclear pharmacokinetic process *in vivo* et al. (39). Therefore, it could be a better strategy to re-evaluate clinically available drugs with known pharmacokinetic and pharmacodynamics profiles, which can be re-purposed as ABCB1-mediated reversal agents of MDR.

NVP-CGM097 is an HDM2 inhibitor that can interrupt the binding of HDM2-p53 to release wild-type p53, thereby activating p53 and leading to cell apoptosis (40). Phase I clinical trials using NVP-CGM097 is now under way for the treatment of WT-p53 solid tumors. Besides, the combination of NVP-CGM097 with other clinically available drugs such as 5-fluoroucracil, temozolomide, or everolimus (RAD001) showed additive antiproliferative effects in neuroendocrine tumor cell line GOT1 cells, in particular, the co-incubation with 5-fluoroucracil also increased p53 and p21 expression in an additive manner (41). In addition, the combination of AEB071 (protein kinase C inhibitors) and NVP-CGM097 resulted in tumor stasis or regression (42). There is currently no research on the effects of NVP-CGM097 on MDR mediated by ABCB1. To our knowledge, this article may be the first to report the inhibition activity of NVP-CGM097 against ABCB1. In this study, we started with the investigation of the toxicity of NVP-CGM097 in ABCB1-overexpressing cells by MTT assay. Based on the proliferation curves of inhibitory rate, we selected the concentration of NVP-CGM097 in which proliferation inhibitory rate was <20% for combinational studies. It was found that the IC_50_ values of doxorubicin and paclitaxel in ABCB1-overexpressing cells was decreased significantly after co-administration with NVP-CGM097, suggesting that NVP-CGM097 can reverse the MDR of drug-resistant cells in a concentration-dependent manner. Subsequently, the mechanism of NVP-CGM097 reversing MDR was studied. Given that ABC transporters are important membrane proteins mainly for uptake and expulsion of a variety of substrates, suppressing the efflux function of ABCB1 transporter may help to increase the concentration of chemotherapeutic drugs in ABCB1-Overexpressing tumor cells, and consequently improving the cell-killing efficacy (43). In this study, [^3^H]-paclitaxel accumulation and efflux assay verified that NVP-CGM097 could boost the intracellular accumulation of ABCB1-substrate chemotherapeutic drugs in ABCB1-overexpressing cells by blocking the efflux function of ABCB1.

The function of ABC transporters is energized by ATPase mediated ATP hydrolysis (44). In our study, NVP-CGM097 stimulated ABCB1-ATPase when the concentration is lower than 10 μM and inhibited ABCB1-associated ATPase at higher concentration of NVP-CGM097. Previous studies have also shown that some ABCB1 inhibitors could either stimulate (45), inhibit (46), or show stimulatory effect at lower concentration while inhibitory effect at higher concentration (47). Such discrimination in ABCB1 inhibitors were explained recently by Alam et al. through a set of substrate- and inhibitor-binding human ABCB1 cryo-EM protein models (35). Thus, we performed molecular docking simulation in both models to further illustrate the interaction. According to our docking results, NVP-CGM097 had good binding affinity in both substrate and inhibitor-binding ABCB1 models. NVP-CGM097 likely occupied substrate-binding pocket thus stimulated ABCB1-associated ATPase at lower concentration, while with the concentration increasing, NVP-CGM097 tended to bind to the inhibitory site, which led to slight but critical conformational changes in the transporter and reduced the ATPase activity. Such conformational change could be the shift of transmembrane domain, resulting in increased distance between nucleotide binding domains (35).

The reversal of ABCB1-mediated MDR can be accomplished either by suppressing the ABCB1 efflux process or down-regulating the ABCB1 expression. This research then explored the impact of NVP-CGM097 on the degree of expression and subcellular localization of ABCB1 protein. The findings indicated that NVP-CGM097 did not affect the protein expression and subcellular localization of ABCB1 upon incubation with NVP-CGM097. Further study is needed whether long time NVP-CGM097 treatment could affect the expression and/or localization of ABCB1.

In conclusion, this study demonstrates that NVP-CGM097 can reverse the MDR in ABCB1-overexpressing cancer cells through blocking the function of ABCB1 transporter. NVP-CGM097 inhibits the efflux function of the ABCB1 transporter, which in turn increases the intracellular accumulation of chemotherapeutic drugs to induce augmented cytotoxicity effects. These results suggest that the usage of NVP-CGM097 would have the potential to be expanded as a combinational therapy with chemotherapeutic drugs in treating solid tumors. Patients bearing ABCB1-overexpressed tumors may benefit from NVP-CGM097 combinational chemotherapy.

## Data Availability Statement

The original contributions presented in the study are included in the article/supplementary material, further inquiries can be directed to the corresponding author/s.

## Author Contributions

ZS-C, X-QL, and JL designed the experiments. MZ and X-YC performed the experiments and wrote the paper. X-DD, WF, J-QW, and Q-XT performed the experiments. QC analyzed the data. All authors read and approved the final manuscript.

## Conflict of Interest

The authors declare that the research was conducted in the absence of any commercial or financial relationships that could be construed as a potential conflict of interest.
